# Supplementary material for: A single dose of an adenovirus-vectored vaccine provides protection against SARS-CoV-2 challenge
Source: Nat Commun. 2020 Aug 14;11:4081. doi: 10.1038/s41467-020-17972-1 (PMC7427994; doi:10.1038/s41467-020-17972-1)
Supplement: Supplementary file 1 — Supplementary Information [file 41467_2020_17972_MOESM1_ESM.pdf]

Supplementary Information for:

A single dose of an adenovirus-vectored vaccine provides protection against SARS-CoV-2 challenge

Shipo Wu [1,3], Gongxun Zhong [2,3], Jun Zhang[1,3], Lei Shuai[2,3], Zhe Zhang[1], Zhiyuan Wen[2], Busen Wang[1], Zhenghao Zhao[1], Xiaohong Song[1], Yi Chen[1], Renqiang Liu[2], Ling Fu[1], Jinlong Zhang[1], Qiang Guo[1], Chong Wang[2], Yilong Yang[1], Ting Fang[1], Peng Lv[1], Jinliang Wang[2], Junjie Xu[1], Jianmin Li[1], Changming Yu[1], Lihua Hou[1]✉, Zhigao Bu[2]✉ ,Wei Chen[1]✉

[1] Beijing Institute of Biotechnology, Beijing, China.

[2] State key Laboratory of Veterinary Biotechnology, Harbin Veterinary Research Institute, Harbin, China.

[3] These authors contributed equally.

✉ Corresponding authors. Email: cw0226@foxmail.com; buzhgao@caas.cn; houlihua@sina.com.

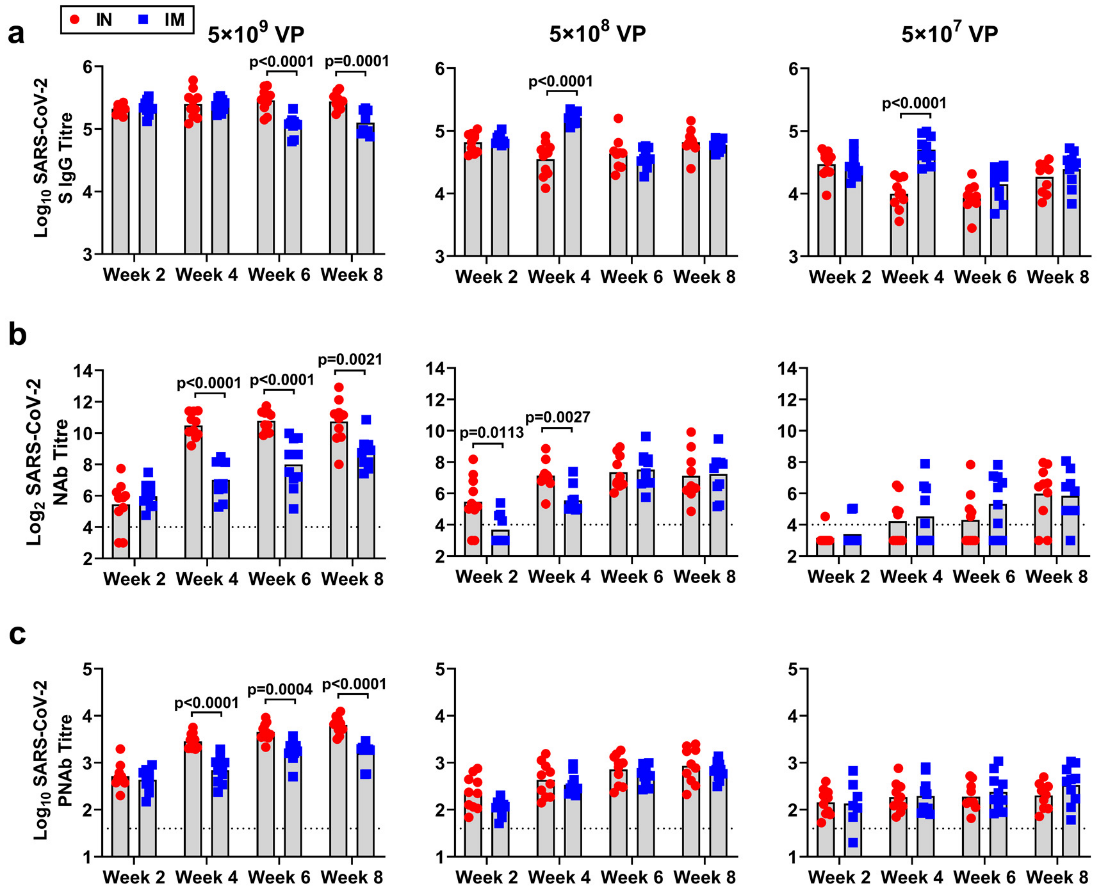

Supplementary Figure 1. Comparison of humoral immune responses to Ad5-nCoV induced by vaccination via the IM and IN routes. BALB/c mice (n = 10 biologically independent animals per group) received a single immunization with 5×10^9 VP, 5×10^8 VP, or 5×10^7 VP of Ad5-nCoV by the IM or IN route, and the humoral immune responses were assessed at weeks 2, 4, 6 and 8 following vaccination by S-specific ELISA, SARS-CoV-2 NAb titration (MN_{50}) and SARS-CoV-2 PNAb titration. a, S-specific serum IgG titres induced by Ad5-nCoV via the IM and IN routes at different doses. b, SARS-CoV-2 NAb titres induced by Ad5-nCoV via the IM and IN routes at different doses. c, SARS-CoV-2 PNAb titres induced by Ad5-nCoV via the IM and IN routes at different doses. Bar at the geometric mean; statistical significance was determined by two-tailed nonparametric Mann–Whitney's rank test; IN route = red circles; IM route = blue squares; dotted line = the limit of detection. Source data are provided as a Source Data file.

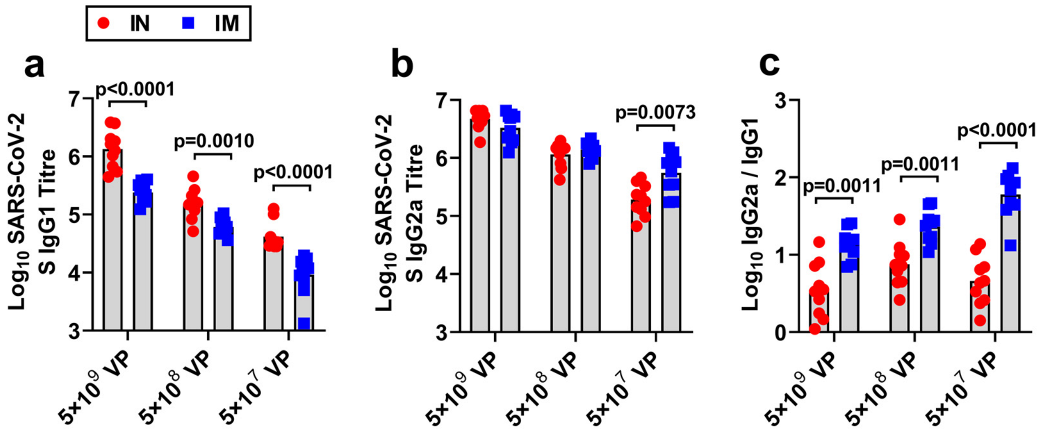

Supplementary Figure 2. SARS-CoV-2 S specific IgG1 and IgG2a responses at week 8 post immunization. SARS-CoV-2 S specific IgG1 and IgG2a titres in serum of the Ad5-nCoV vaccinated mice (n = 10 biologically independent animals per group) at week 8 post immunization had been detected. a, SARS-CoV-2 S specific IgG1 titres induced by Ad5-nCoV via the IM and IN routes at different doses. b, SARS-CoV-2 S specific IgG2a titres induced by Ad5-nCoV via the IM and IN routes at different doses. c, The ratio of IgG2a to IgG1 in groups of the IM and IN vaccination mice. Bar at the geometric mean; statistical significance was determined by two-tailed nonparametric Mann–Whitney's rank test; IN route = red circles; IM route = blue squares. Source data are provided as a Source Data file.

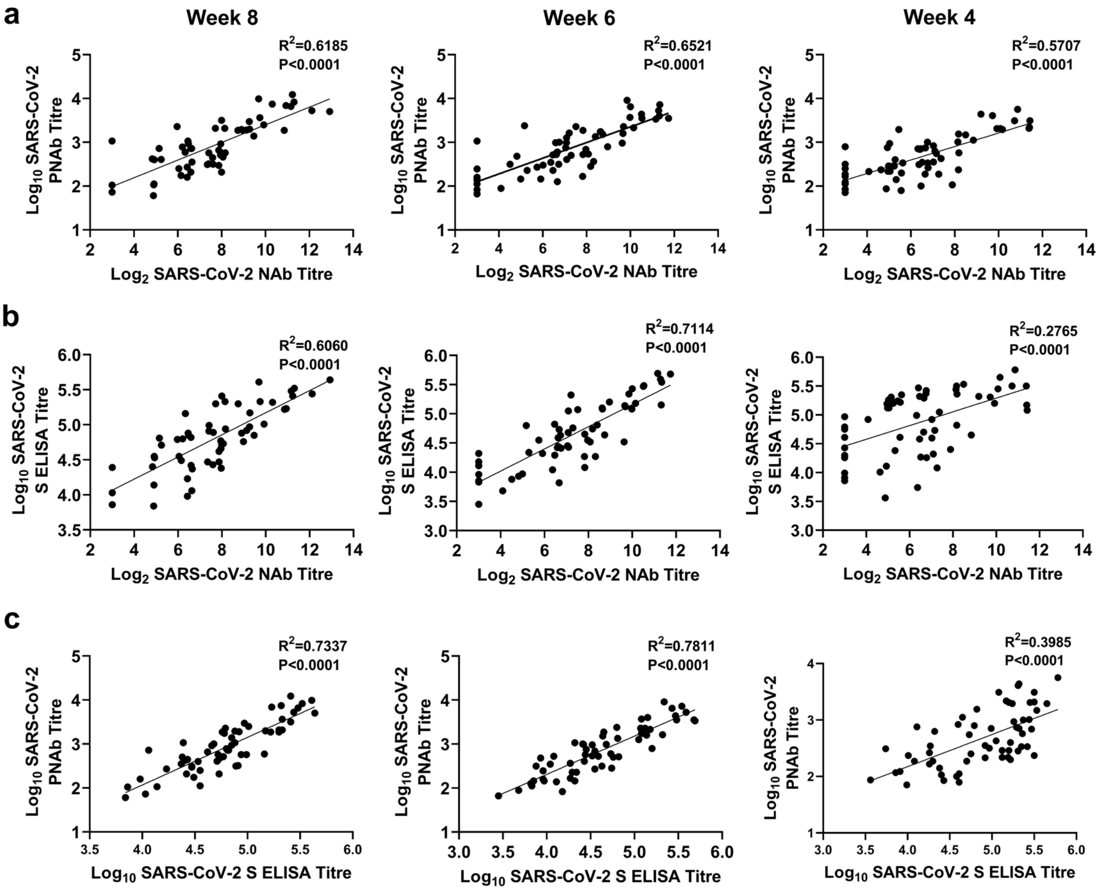

Supplementary Figure 3. Correlates of S-specific IgG titres, SARS-CoV-2 NAb titres and SARS-CoV-2 PNAb titres in Ad5-nCoV-immunized mice. Pearson's correlation coefficient was calculated among S-specific IgG titres, SARS-CoV-2 NAb titres and SARS-CoV-2 PNAb titres in Ad5-nCoV-immunized mice at weeks 4, 6 and 8 post immunization. a, Correlates of SARS-CoV-2 NAb titres and PNAb titres. b, Correlates of S-specific IgG titres and SARS-CoV-2 NAb titres. c, Correlates of S-specific IgG titres and SARS-CoV-2 PNAb titres. Source data are provided as a Source Data file.

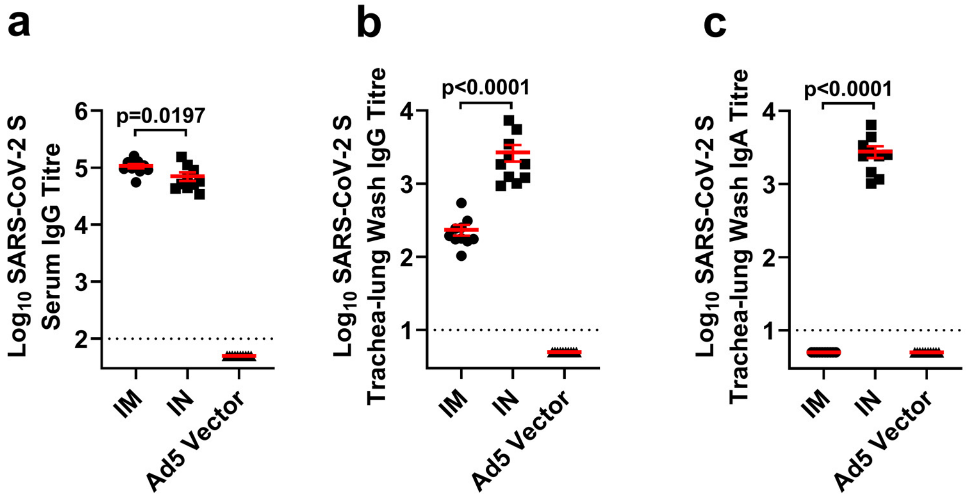

Supplementary Figure 4. IgG and IgA responses in the trachea-lung washes of Ad5-nCoV-immunized mice. BALB/c mice (n = 10 biologically independent animals per group) received a single immunization with 5×10^8 VP of Ad5-nCoV by the IM or IN route or were immunized with Ad5 vector as a control. At 2 weeks after immunization, mice were euthanized, splenocytes were prepared to analyse the cellular immune response (Fig. 1i, 1j), and serum and trachea-lung washes were collected for IgG and IgA detection. a, Serum S-specific IgG titres in immunized and control mice. b, Trachea-lung wash S-specific IgG titres in immunized and control mice. c, Trachea-lung wash S-specific IgA titres in immunized and control mice. Data are presented as mean ± s.e.m. Statistical significance was determined by two-tailed nonparametric Mann–Whitney's rank test. Dotted line = the limit of detection. Source data are provided as a Source Data file.

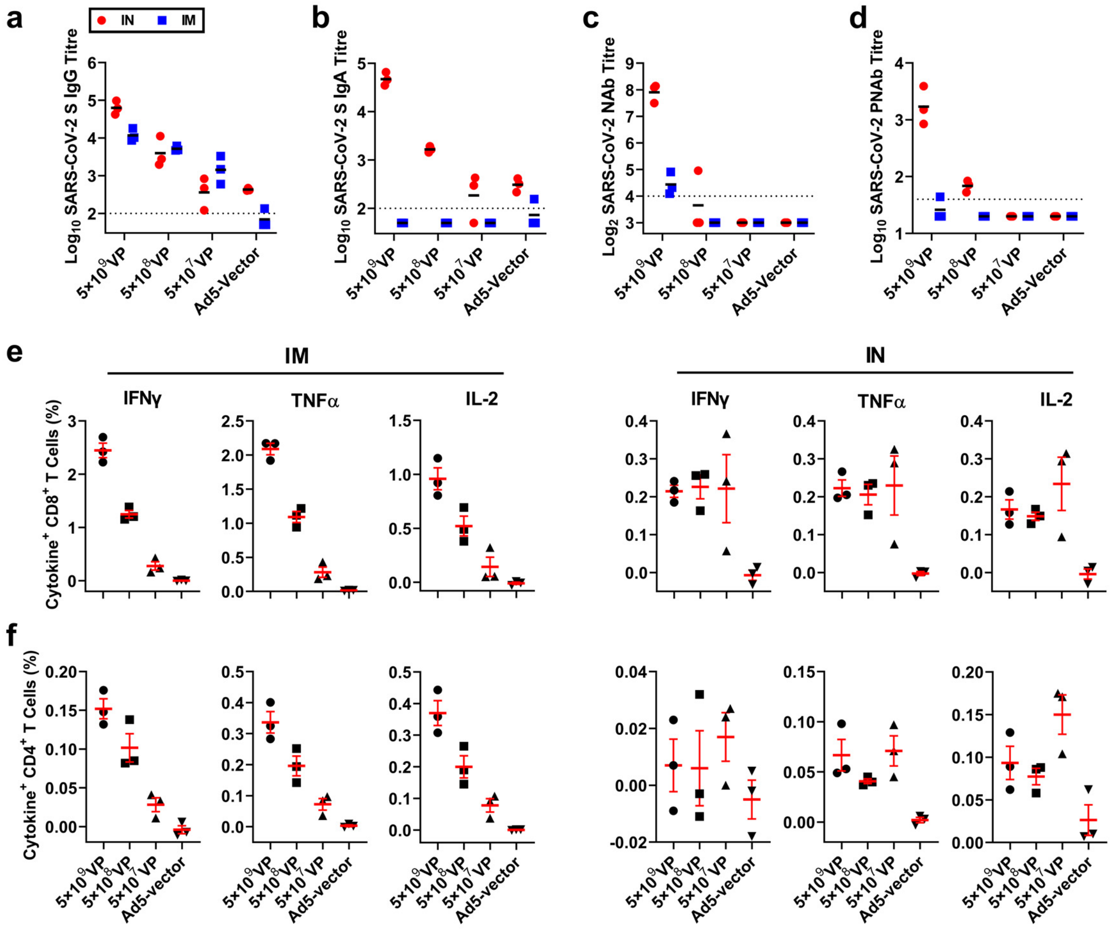

Supplementary Figure 5. The humoral immune responses in the trachea-lung washes and splenic cellular immune responses at week 10 post immunization. BALB/c mice (n = 10 biologically independent animals per group) received a single immunization with different doses of Ad5-nCoV or Ad5 vector by the IM or IN route, seven of ten mice were challenged at week 10, and the remaining three mice were euthanized for immunogenicity detection. a-d, Humoral immune responses were detected in trachea-lung washes for S-specific IgG titres (a), S-specific IgA titres (b), SARS-CoV-2 NAb titres (c) and SARS-CoV-2 PNAb titres (d) with n = 3 biologically independent animals per group. Black bars reflect the geometric means. e, f, Splenocytes were prepared for intracellular cytokine staining, and S-specific IFNγ, TNFα, and IL-2 were detected in $CD8^+$ T cells (e) and $CD4^+$ T cells (f) with n = 3 biologically independent animals per group. Data are presented as mean ± s.e.m. IN route = red circles; IM route = blue squares; dotted line = the limit of detection. Source data are provided as a Source Data file.

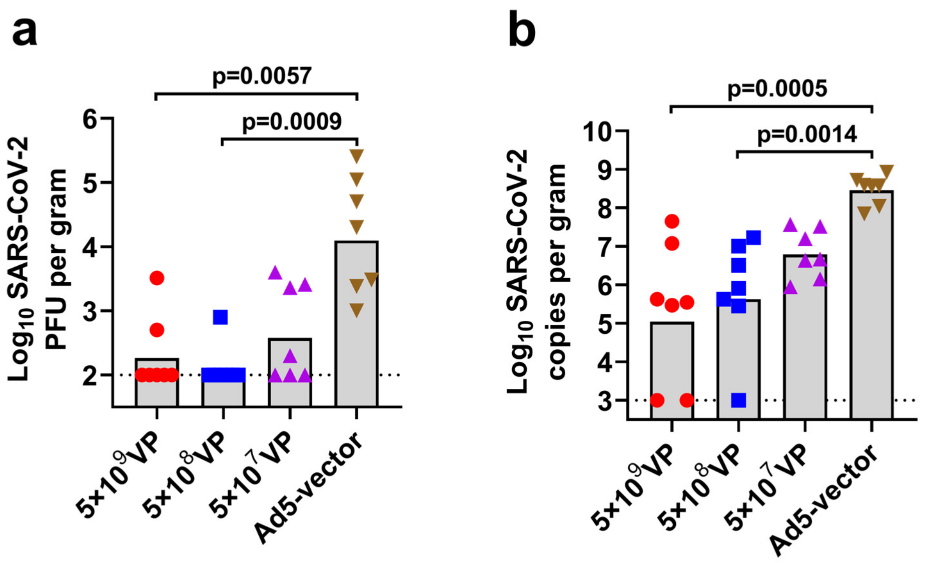

Supplementary Figure 6. Turbinate viral loads in mice vaccination via IM route. a, Turbinate live virus numbers (PFU / g) in different IM vaccination groups after mouse-adapted SARS-CoV-2 challenge with n = 7 biologically independent animals per group. b, Turbinate viral loads (copies / g) in different IM vaccination groups after challenge with n = 7 biologically independent animals per group. Bar at the geometric mean, statistical significance was determined by Kruskal-Wallis ANOVA with Dunn's multiple comparisons tests. Source data are provided as a Source Data file.

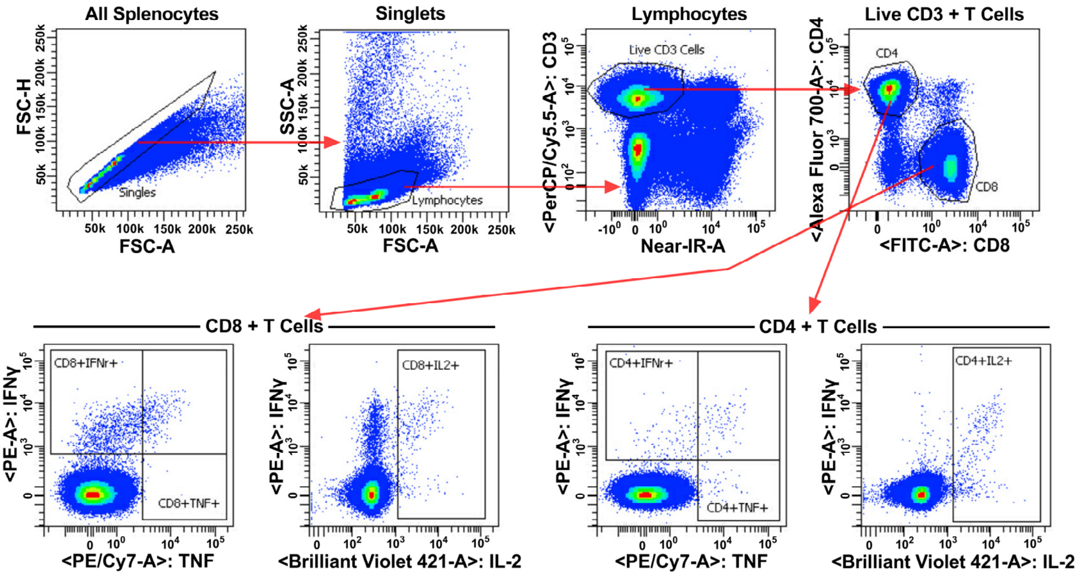

Supplementary Figure 7. Gating strategy of intracellular cytokine staining flow cytometry. Stimulated cells were stained and analyzed by flow cytometry. The sample was progressively gated to identify single cells, lymphocytes, live CD3 + T cells, and CD4 or CD8 + T cells as shown in the top row. Within CD4 or CD8 + T cells, gates for IFNγ, TNF and IL-2 were created (bottom).

Supplementary Table 1. Primers used for sequencing of SARS-CoV-2 spike protein and qPCR.

Name	Sequence	Application
pDC316-F	5’-ACG TGG GTA TAA GAG GCG-3’	sequencing
pDC316-R	5’-CGA TGC TAG ACG ATC CAG-3’	sequencing
SG-1	5’-ACT TCA AGA ACC TCC GGG A-3’	sequencing
SG-2	5’-ACC AAG CTG AAC GAT CTG TG-3’	sequencing
SG-3	5’-CTG GAC ATC ACA CCC TGC AG-3’	sequencing
SG-4	5’-GTG AAG CAG ATC TAC AAG AC-3’	sequencing
SG-5	5’-GAC AAG GTG GAG GCC GAG GT-3’	sequencing
SG-6	5’-AGG CTG AAC GAG GTG GCC AA-3’	sequencing
N-forward	5’-GGG GAA CTT CTC CTG CTA GAA T-3’	qPCR
N-reverse	5'-CAG ACA TTT TGC TCT CAA GCT G-3'	qPCR
Probe	5'-FAM-TTG CTG CTG CTT GAC AGA TT-TAMRA-3'	qPCR
