# Supplementary material for: A single dose of an adenovirus-vectored vaccine provides protection against SARS-CoV-2 challenge
Source: Nat Commun. 2020 Aug 14;11:4081. doi: 10.1038/s41467-020-17972-1 (PMC7427994; doi:10.1038/s41467-020-17972-1)
Supplement: Supplementary file 3 — Reporting Summary [file 41467_2020_17972_MOESM3_ESM.pdf]

nature research

Corresponding author(s): Prof. Wei Chen

Last updated by author(s): Jul 22, 2020

Reporting Summary

Nature Research wishes to improve the reproducibility of the work that we publish. This form provides structure for consistency and transparency in reporting. For further information on Nature Research policies, see our Editorial Policies and the Editorial Policy Checklist.

Statistics

For all statistical analyses, confirm that the following items are present in the figure legend, table legend, main text, or Methods section.

n/a	Confirmed	
☐	☒	The exact sample size (n) for each experimental group/condition, given as a discrete number and unit of measurement
☐	☒	A statement on whether measurements were taken from distinct samples or whether the same sample was measured repeatedly
☐	☒	The statistical test(s) used AND whether they are one- or two-sided<br>*Only common tests should be described solely by name; describe more complex techniques in the Methods section.*
☒	☐	A description of all covariates tested
☐	☒	A description of any assumptions or corrections, such as tests of normality and adjustment for multiple comparisons
☐	☒	A full description of the statistical parameters including central tendency (e.g. means) or other basic estimates (e.g. regression coefficient) AND variation (e.g. standard deviation) or associated estimates of uncertainty (e.g. confidence intervals)
☐	☒	For null hypothesis testing, the test statistic (e.g. F, t, r) with confidence intervals, effect sizes, degrees of freedom and P value noted<br>*Give P values as exact values whenever suitable.*
☒	☐	For Bayesian analysis, information on the choice of priors and Markov chain Monte Carlo settings
☒	☐	For hierarchical and complex designs, identification of the appropriate level for tests and full reporting of outcomes
☐	☒	Estimates of effect sizes (e.g. Cohen's d, Pearson's r), indicating how they were calculated

Our web collection on statistics for biologists contains articles on many of the points above.

Software and code

Policy information about availability of computer code

Data collection	Flow cytometry date collected using BD FACSDiva v.8.0.1 software associated with the BD FACS CantoTM flow cytometer.
Data analysis	The full spike protein gene of SARS-CoV-2 was codon optimized by UpGene v5.5.0.3 software.<br>Flow cytometry data were analyzed using BD FACSDiva v.8.0.1.<br>All the statistical analysis were performed using GraphPad Prism v8.0.2. Graphs and plots were also generated using GraphPad Prism v8.0.2.<br>All the statistical analyses are two-sided. All correlations are rank-based spearman's correlations in which r represent Spearman's r and p represent two-tailed p values.

For manuscripts utilizing custom algorithms or software that are central to the research but not yet described in published literature, software must be made available to editors and reviewers. We strongly encourage code deposition in a community repository (e.g. GitHub). See the Nature Research guidelines for submitting code & software for further information.

Data

Policy information about availability of data

All manuscripts must include a data availability statement. This statement should provide the following information, where applicable:

- Accession codes, unique identifiers, or web links for publicly available datasets
- A list of figures that have associated raw data
- A description of any restrictions on data availability

The authors declare that the data supporting the findings of this study are available within the paper and its supplementary information files, the source data are provided as a Source Data file. Source data are provided with this paper.

Field-specific reporting

Please select the one below that is the best fit for your research. If you are not sure, read the appropriate sections before making your selection.

☒ Life sciences ☐ Behavioural & social sciences ☐ Ecological, evolutionary & environmental sciences

For a reference copy of the document with all sections, see nature.com/documents/nr-reporting-summary-flat.pdf

Life sciences study design

All studies must disclose on these points even when the disclosure is negative.

Sample size	No statistical method was used to determine the sample size. Sample size were selected based on our previous vaccine studies in these animal models, and ability to detect statistical differences between experimental groups of this size. The sample size for each experiment is indicated in the figure legend for each experiment.
Data exclusions	Several humoral response data in mice model were missed due to the insufficient of the serum when relevant tests were carried out. Those are IgG titre of mice No. 5 in 5×10e8 VP dose group via IN route at week 6, NAb titres of mice No. 6 in 5×10e7 VP dose group via IM route at week 8 and mice No. 2 in 5×10e8 VP dose group via IN route at week 8, and PNAb titres of mice No. 1, 6 and 9 in 5×10e7 VP dose group via IM route at week 2.
Replication	Ten mice and six ferrets were included for each vaccination and control group. As for the viral loads, viral titers, at least 3 mice for each group (at each detecting time-point) were tested. Some tests were repeated twice separately by two technicians. All attempts at replication were successful.
Randomization	Animals were randomly allocated based on age, body weight and sex.
Blinding	The investigators were not blinded to group allocation during Ad5-nCoV vaccination and collection of specimens from animals. However, the investigators were blinded to group allocation during data acquisition / analysis such as the measurements of serum or trachea-lung wash IgG, IgA, NAb or PNAb titres, lung, turbinate or nasal wash viral loads and splenic cellular immune response.

Reporting for specific materials, systems and methods

We require information from authors about some types of materials, experimental systems and methods used in many studies. Here, indicate whether each material, system or method listed is relevant to your study. If you are not sure if a list item applies to your research, read the appropriate section before selecting a response.

Materials & experimental systems

n/a	Involved in the study
☐	☒ Antibodies
☐	☒ Eukaryotic cell lines
☒	☐ Palaeontology and archaeology
☐	☒ Animals and other organisms
☒	☐ Human research participants
☒	☐ Clinical data
☒	☐ Dual use research of concern

Methods

n/a	Involved in the study
☒	☐ ChIP-seq
☐	☒ Flow cytometry
☒	☐ MRI-based neuroimaging

Antibodies

Antibodies used

FITC anti-mouse CD8 (Clone 5H10-1, Biolegend, Cat: 100804, Lot: B201801) 0.25 in 50 μL,
PE anti-mouse IFNγ (Clone XMG1.2, BD, Cat: 554412, Lot: 7081524) 0.5 in 50 μL,
PerCP/Cy5.5 anti-mouse CD3 (Clone 17A2, BD, Cat: 560527, Lot: 7208643) 0.15 in 50 μL,
PE/Cy7 anti-mouse TNF (Clone MP6-XT22, BD, Cat: 557644, Lot: 7235503) 0.5 in 50 μL,
Alexa Fluor® 700 anti-mouse CD4 (Clone RM4-5, Biolegend, Cat: 100536, Lot: B248742) 0.15 in 50 μL,
Brilliant Violet 421TM anti-mouse IL2 (Clone JES6-5H4, Biolegend, Cat: 503826, Lot: B242079) 0.3 in 50 μL,
HRP-conjugated goat anti-mouse IgG (Abcam, Cat: ab97265, Lot: GR3195192-8) 1 in 10,000 μL,
HRP-conjugated goat anti-mouse IgG1 (Abcam, Cat: ab97240, Lot: GR168230-1) 1 in 10,000 μL,
HRP-conjugated goat anti-mouse IgG2a (Abcam, Cat: ab97245, Lot: GR174542-3) 1 in 10,000 μL,
HRP-conjugated goat anti-mouse IgA (Abcam, Cat: ab97235, Lot: GR157829) 1 in 10,000 μL,
Horseradish peroxidase (HRP)-conjugated goat anti-rabbit IgG antibody (Cell Signaling Technology, Cat: 7074P2, Lot: 26) 1 in 10,000 μL,
HRP-conjugated anti-β-actin antibody (Abcam, Cat: ab49900, Lot: GR276781-1) 1 in 10,000 μL,
Polyclonal rabbit anti-SARS-CoV spike antibody (Sino Biological, Cat: 40150-t52, Lot: HD09SE0630-B) 1 in 2,000 μL.

April 2020

Validation

All commercially available antibodies used are validated.

ELISA
HRP-conjugated goat anti-mouse IgG (Abcam, Cat: ab97265): specific for mouse IgG, suitable for ICC, IHC-P, ELISA and WB, Manufacturer website provides the datasheet "Goat Anti-Mouse IgG Fc (HRP) ab97265", The datasheet states this antibody was used in 23 citations.
HRP-conjugated goat anti-mouse IgG1 (Abcam, Cat: ab97240): specific for mouse IgG1, suitable for ICC, ELISA, IHC-P and WB, Manufacturer website provides the datasheet "Goat Anti-Mouse IgG1 (HRP) ab97240", The datasheet states this antibody was used in 49 citations.
HRP-conjugated goat anti-mouse IgG2a (Abcam, Cat: ab97245): specific for mouse IgG2a, suitable for IHC-P, ELISA, WB and ICC, Manufacturer website provides the datasheet "Goat Anti-Mouse IgG2a heavy chain (HRP) ab97245", The datasheet states this antibody was used in 24 citations.
HRP-conjugated goat anti-mouse IgA (Abcam, Cat: ab97235): specific for mouse IgA, suitable for ICC, IHC-P, ELISA and WB, Manufacturer website provides the datasheet "Goat Anti-Mouse IgA alpha chain (HRP) ab97235", The datasheet states this antibody was used in 11 citations.

Western blotting
Horseradish peroxidase (HRP)-conjugated goat anti-rabbit IgG antibody (Cell Signaling Technology, Cat: 7074P2): specific for rabbit IgG, suitable for WB, IP, IHC, ChIP, IF, FC, Manufacturer website provides the datasheet "Anti-rabbit IgG, HRP-linked Antibody", The datasheet states this antibody was used in 5632 citations.
HRP-conjugated anti-β-actin antibody (Abcam, Cat: ab49900): Reaction with a wide range of species, including Mouse, Rat, Sheep, Rabbit, Chicken, Guinea pig, Hamster, Cow, Cat, Dog, Human, Pig, Carp, Monkey, etc; suitable for WB, Manufacturer website provides the datasheet "Anti-beta Actin antibody [AC-15] (HRP) ab49900", The datasheet states this antibody was used in 213 citations.
Polyclonal rabbit anti-SARS-CoV spike antibody (Sino Biological, Cat: 40150-t52): specific for SARS Coronavirus Spike Protein, suitable for WB, ELISA, FCM and IP, Manufacturer website provides the datasheet "Human SARS Coronavirus Spike Antibody, Rabbit PAb, Antigen Affinity Purified".

Flow Cytometry
FITC anti-mouse CD8 (Clone 5H10-1, Biolegend, Cat: 100804): Reactivity, Mouse; Application, Flow cytometry (Quality tested); this antibody was used in 6 citations.
PE anti-mouse IFNγ (Clone XMG1.2, BD, Cat: 554412): Reactivity, Mouse; Application, Intracellular staining (flow cytometry) (Routinely Tested); this antibody was used in more than 10 citations.
PerCP/Cy5.5 anti-mouse CD3 (Clone 17A2, BD, Cat: 560527): Reactivity, Mouse; Application, Flow cytometry (Routinely Tested); this antibody was used in more than 10 citations.
PE/Cy7 anti-mouse TNF (Clone MP6-XT22, BD, Cat: 557644): Reactivity, Mouse; Application, Intracellular staining (flow cytometry) (Routinely Tested), this antibody was used in more than 10 citations.
Alexa Fluor® 700 anti-mouse CD4 (Clone RM4-5, Biolegend, Cat: 100536): Reactivity, Mouse; Application, Flow cytometry (Quality tested); this antibody was used in more than 10 citations.
Brilliant Violet 421TM anti-mouse IL2 (Clone JES6-5H4, Biolegend, Cat: 503826): Reactivity, Mouse; Application, Flow cytometry (Quality tested); this antibody was used in 4 citations.

Eukaryotic cell lines

Policy information about cell lines

Cell line source(s)

HEK293 cells: ATCC, CRL-1573
Vero E6 cells: ATCC, CRL-1586
ACE2-293T cells: Constructed by hygromycin B screening, from 293 T cells (ATCC, CRL-11268)

Authentication

To follow the protocol provided by ATCC or Thermo Scientific. None of the cell lines have been authenticated.

Mycoplasma contamination

The cell line tested negative for mycoplasma contamination by MycoAlertTM Mycoplasma Detection Kit (Rockland, ME, USA).

Commonly misidentified lines
(See ICLAC register)

There is no commonly misidentified cell lines used in this study.

Animals and other organisms

Policy information about studies involving animals; ARRIVE guidelines recommended for reporting animal research

Laboratory animals

Specific pathogen-free (SPF) female BALB/c mice aged 6-8 weeks were obtained from Beijing Vital River Laboratory Animal Technologies Co., Ltd. (Beijing, China). Three- to four-month-old female Angora ferrets purchased from Wuxi Cay Ferret Farm (Wuxi, China). The mice were housed in a temperature-, humidity- and light cycle-controlled facility (20°C ± 2°C; 50% ± 10 %; light, 7:00-19:00; dark, 19:00–7:00).

Wild animals

Study did not involve wild animals.

Field-collected samples

Study did not involve samples collected from the field.

Ethics oversight	For immunogenicity test in mice model, the procedure was reviewed and approved by the Institutional Experimental Animal Welfare and Ethics Committee of Beijing Institute of Biotechnology. For SARS-CoV-2 challenge in mice and ferret model, the procedure was reviewed and approved by Harbin Veterinary Research Institute, Chinese Academy of Agricultural Sciences.

Note that full information on the approval of the study protocol must also be provided in the manuscript.

Flow Cytometry

Plots

Confirm that:

- [x] The axis labels state the marker and fluorochrome used (e.g. CD4-FITC).
- [x] The axis scales are clearly visible. Include numbers along axes only for bottom left plot of group (a 'group' is an analysis of identical markers).
- [x] All plots are contour plots with outliers or pseudocolor plots.
- [x] A numerical value for number of cells or percentage (with statistics) is provided.

Methodology

Sample preparation	Under aseptic conditions, Spleens were pushed through a 70-μm cell strainer in complete RPMI1640 medium to prepare a single cell suspension. Splenocytes were centrifuged at 500 × g for 5 min, the supernatant was discarded, and red blood cells removed with ACK lysing buffer (0.15 M of NH4Cl, 10 mM of KHCO3, 0.1 mM of Na2EDTA, pH 7.2–7.4). Cells were washed twice in complete RPMI 1640 medium, counted, and diluted to 4×106 cells per mL. The cells (2 million per tupe) were stimulated for 6 h at 37 °C with or without 1 μg/mL of overlapping 15-amino-acid peptides covering the S protein and with BD GolgiStopTM and BD GolgiPlugTM to block cytokine secretion. Following peptide pool stimulation, the splenocytes were washed and stained with a mixture of antibodies against lineage markers, including anti-CD3 PerCP-Cy5.5 (clone 17A2), anti-CD4 Alexa Fluor 700 (clone RM4-5), and anti-CD8 FITC (clone 5H10-1), and the viability dye Near-IR to exclude dead cells from data analysis. After one wash with PBS, the cells were fixed and permeabilized with Cytofix/Cytoperm (BD Biosciences, USA), washed with Perm/Wash buffer (BD Biosciences, USA), and stained with anti-IFNγ PE (clone XMG1.2), anti-TNF PE-Cy7 (clone MP6-XT22) and anti-IL-2 Brilliant Violet 421 (clone JES6-5H4). The cells were washed successively with Perm/Wash buffer and PBS and resuspended in PBS.
Instrument	BD FACS CantoTM
Software	BD FACSDiva V8.0.1
Cell population abundance	At least 2 million splenocytes per sample were used for intracellular cytokine staining, and at least 200,000 events were collected for each sample on flow cytometer.
Gating strategy	Live CD4 or CD8 T cells were selected in four straightforward steps.<br>1) Singlets obtained using FSC-A vs FSC-H.<br>2) Lymphocytes obtained using FSC-A vs SSC-A.<br>3) Live T cells were selected as Near-IR- CD3+.<br>4) CD8 and CD4 T cells were selected as CD3+CD8+ or CD3+CD4+.

- [x] Tick this box to confirm that a figure exemplifying the gating strategy is provided in the Supplementary Information.
